# Supplementary material for: Wheat Oxylipins in Response to Aphids, CO2 and Nitrogen Regimes
Source: Molecules. 2023 May 16;28(10):4133. doi: 10.3390/molecules28104133 (PMC10221955; doi:10.3390/molecules28104133)
Supplement: Supplementary file 1 [file molecules-28-04133-s001.zip › molecules-2232597-supplementary.pdf]

# Supplementary Material

**Table S1**.Qualitative profile of phytoprostanes in terms of their occurrence and distribution in plant physiological part, fruits and processed plant foods.

| Plant /Food Sample | 9-F1t-PhytoP | 9-*epi*-9-F1t-PhytoP | 9-D1t-PhytoP | 9-*epi*-9-D1t-PhytoP | 9-L1-PhytoP | 16-B1-PhytoP | *ent*-16-F1t-PhytoP + *ent*-16-*epi*-16-F1t-PhytoP | Reference |
|---|---|---|---|---|---|---|---|---|
| *Plant Physiological Parts* | | | | | | | | |
| **Wheat leaves** | ✓ | ✓ | | | | | ✓ | **this study** |
| Cucumis melo L. leaves | ✓ | ✓ | | | ✓ | ✓ | ✓ | [42] |
| Date tree leaves | ✓ | ✓ | ✓ | ✓ | ✓ | ✓ | | [43,44] |
| Chilean hazelnut (Gevuina avellana Mol., Proteaceae) cotyledons | ✓ | ✓ | ✓ | ✓ | | ✓ | ✓ | [47] |
| Macroalgae | ✓ | ✓ | | | ✓ | ✓ | | [45] |
| Brown macroalgae (Ectocarpus siliculosus) | ✓ | ✓ | | | ✓ | ✓ | ✓ | [46] |
| Brown macroalgae (Laminaria digitate) | ✓ | ✓ | | | ✓ | ✓ | ✓ | [46] |
| Brown macroalgae (Fucus spiralis) | ✓ | ✓ | | | ✓ | ✓ | ✓ | [46] |
| Red macroalgae (Osmundea pinnatifida) | ✓ | ✓ | | | | | ✓ | [46] |
| Red macroalgae (Grateloupia turuturu) | ✓ | ✓ | | | | | ✓ | [46] |
| Brown macroalgae (Pelvetia canaliculata) | ✓ | ✓ | | | ✓ | ✓ | ✓ | [46] |
| Passiflora edulis Sims shell | ✓ | ✓ | ✓ | ✓ | ✓ | ✓ | | [18] |
| Passiflora tripartita var.mollisima shell | ✓ | ✓ | ✓ | ✓ | ✓ | ✓ | ✓ | [48] |

| | | | | | | | | |
|---|---|---|---|---|---|---|---|---|
| Physalis peruviana calyx | ✓ | ✓ | ✓ | ✓ | ✓ | ✓ | ✓ | [49] |
| Date tree skin | ✓ | ✓ | ✓ | ✓ | ✓ | ✓ | ✓ | [43,44] |
| Date tree pits | ✓ | ✓ | ✓ | ✓ | ✓ | ✓ | ✓ | [43,44] |
| Date tree pulp | ✓ | ✓ | ✓ | ✓ | ✓ | ✓ | ✓ | [43,44] |
| Date tree cluster | ✓ | ✓ | ✓ | ✓ | ✓ | ✓ | ✓ | [43,44] |
| Date tree pollen | ✓ | ✓ | ✓ | ✓ | ✓ | ✓ | ✓ | [43,44] |
| Cocoa pod husk | ✓ | ✓ | | | ✓ | ✓ | ✓ | [52] |
| Cocoa husk | ✓ | ✓ | | | ✓ | ✓ | ✓ | [52] |
| *Fruits and seeds* | | | | | | | | |
| Olive fruit | ✓ | ✓ | | | ✓ | ✓ | | [36] |
| Almond kernel | ✓ | ✓ | ✓ | ✓ | ✓ | ✓ | ✓ | [22,29] |
| Walnut kernel | ✓ | ✓ | | | ✓ | ✓ | | [28] |
| Pecan kernel | ✓ | ✓ | | | ✓ | ✓ | ✓ | [28] |
| Rice grain | ✓ | ✓ | ✓ | ✓ | ✓ | ✓ | ✓ | [23] |
| Pistachio kernel | ✓ | ✓ | ✓ | ✓ | ✓ | ✓ | ✓ | [20] |
| Macadamia kernel | ✓ | ✓ | | | ✓ | ✓ | ✓ | [28] |
| Cocoa bean | ✓ | ✓ | ✓ | ✓ | ✓ | ✓ | ✓ | [53] |
| Pea (Pisum sativumL.) seed | ✓ | ✓ | ✓ | ✓ | ✓ | ✓ | ✓ | [38,51] |
| French beans (Phaseolus vulgarisL. seed | ✓ | ✓ | ✓ | ✓ | ✓ | ✓ | ✓ | [51] |
| Mangetout (Pisum sativumL. Ssp.arvense) seed | ✓ | ✓ | ✓ | ✓ | ✓ | ✓ | ✓ | [51] |
| Coffee pulp | ✓ | ✓ | | | ✓ | ✓ | ✓ | [52] |
| *Processed Plant Foods* | | | | | | | | |
| Extra virgin olive oil | ✓ | ✓ | ✓ | ✓ | ✓ | ✓ | ✓ | [21,31,35] |
| Olive oil | | | ✓ | ✓ | | | ✓ | [31,32] |
| Argan oil | ✓ | ✓ | | | ✓ | | | [32] |
| Sesame oil | ✓ | | | | ✓ | | ✓ | [32] |
| Grapefruit oil | ✓ | ✓ | | | ✓ | | | [32] |
| Safflower oil | ✓ | | | | ✓ | | ✓ | [32] |
| Palm oil | | | | | ✓ | | | [32] |
| Sunflower oil | ✓ | ✓ | ✓ | ✓ | ✓ | ✓ | ✓ | [31] |
| Olive (treated flesh) | ✓ | ✓ | | | | | | [36] |
| Macadamia kernel fried | ✓ | ✓ | ✓ | ✓ | ✓ | ✓ | ✓ | [28] |
| Pecan kernel fried | ✓ | ✓ | | | ✓ | ✓ | ✓ | [28] |
| Grape must | ✓ | | ✓ | ✓ | | | ✓ | [33] |
| Wine | ✓ | | ✓ | ✓ | ✓ | ✓ | ✓ | [33] |
| Dark chocolate | ✓ | ✓ | ✓ | ✓ | ✓ | ✓ | ✓ | [54] |
| Sea buckthorn juice | ✓ | ✓ | ✓ | ✓ | ✓ | ✓ | ✓ | [34] |

**Table S2:** Qualitative profile of phytofurans in terms of their occurrence and distribution in plant physiological part, fruits and processed plant foods.

| Plant/Food Sample | *ent*-16(*RS*)-9-*epi*-ST-$\Delta^{14}$-10-PhytoF | *ent*-9(*RS*)-12-*epi*-ST-$\Delta^{10}$-13-PhytoF | *ent*-16(*RS*)-13-*epi*-ST-$\Delta^{14}$-9-PhytoF | Reference |
|---|---|---|---|---|
| *Plant Physiological Parts* | | | | |
| **Wheat leaves** | ✓ | ✓ | ✓ | **this study** |
| Cucumis melo L. leaves | ✓ | ✓ | | [42] |
| Date tree leaves | ✓ | ✓ | ✓ | [43,44] |
| Chilean hazelnut (Gevuina avellana Mol., Proteaceae) cotyledons | ✓ | ✓ | ✓ | [47] |
| Brown macroalgae (Ectocarpus siliculosus) | ✓ | ✓ | ✓ | [46] |
| Brown macroalgae (Laminaria digitate) | ✓ | ✓ | ✓ | [46] |
| Brown macroalgae (Pelvetia canaliculata) | ✓ | | | [46] |
| Red macroalgae (Osmundea pinnatifida) | | | ✓ | [6] |
| Red macroalgae (Grateloupia turuturu) | ✓ | | ✓ | [46] |
| Brown macroalage (Fucus spiralis) | ✓ | ✓ | ✓ | [46] |
| Date tree skin | ✓ | ✓ | ✓ | [43] |
| Date tree pits | ✓ | ✓ | ✓ | [43] |
| Date tree pulp | ✓ | ✓ | ✓ | [43] |
| Date tree cluster | ✓ | ✓ | ✓ | [43] |
| Date tree pollen | ✓ | ✓ | ✓ | [43] |
| Cocoa pod husk | ✓ | ✓ | ✓ | [52] |
| Cocoa husk | ✓ | ✓ | ✓ | [52] |
| *Fruits and seeds* | | | | |
| Flax seeds | | | ✓ | [19] |
| Chia seeds | | | ✓ | [19] |
| Cocoa bean | ✓ | ✓ | ✓ | [53] |
| Pea (Pisum sativumL.) | ✓ | ✓ | ✓ | [38,51] |

| | | | | |
|---|---|---|---|---|
| French beans (Phaseolus vulgarisL. | ✓ | ✓ | ✓ | [51] |
| Mangetout (Pisum sativumL. Ssp.arvense) | ✓ | ✓ | ✓ | [51] |
| Rice | ✓ | ✓ | ✓ | [37,50] |
| Almond | ✓ | ✓ | ✓ | [22] |
| Pistachio kernels | ✓ | ✓ | ✓ | [20] |
| Coffee pulp | ✓ | ✓ | ✓ | [52] |
| *Processed Plant Foods* | | | | |
| Extra virgin olive oil | ✓ | ✓ | ✓ | [32] |
| Flax oil | ✓ | ✓ | ✓ | [32] |
| Argan oil | | | ✓ | [32] |
| Sesame oil | | | ✓ | [32] |
| Grapeseed oil | | ✓ | ✓ | [32] |
| Safflower oil | | | ✓ | [32] |
| Palm oil | | ✓ | ✓ | [32] |
| Dark chocolate | ✓ | | ✓ | [54] |
| Sea buckthorn juice | | ✓ | | [34] |
